# Diagnostic performance of 3D-multi-Echo-data-image-combination (MEDIC) for evaluating SLAP lesions of the shoulder

**DOI:** 10.1186/s12891-019-2986-1

**Published:** 2019-12-12

**Authors:** Felix Wuennemann, Laurent Kintzelé, Felix Zeifang, Michael W. Maier, Iris Burkholder, Marc-André Weber, Hans-Ulrich Kauczor, Christoph Rehnitz

**Affiliations:** 1 Diagnostic and Interventional Radiology, University Hospital Heidelberg, Im Neuenheimer Feld 110, 69120 Heidelberg, Germany; 2 Center for Orthopedics, Trauma Surgery and Spinal Cord Injury, University Hospital Heidelberg, Schlierbacher Landstraße 200A, 69118 Heidelberg, Germany; 3 Swabian Joint Center Stuttgart, ATOS Clinic Stuttgart, Hohenheimer Straße 91, 70184 Stuttgart, Germany; 4 Department of Nursing and Health, University of Applied Sciences of the Saarland, Malstatter Straße 17, 66117 Saarbruecken, Germany; 5 Institute of Diagnostic and Interventional Radiology, Pediatric Radiology and Neuroradiology, University Medical Center Rostock, Ernst-Heydemann-Straße 6, 18057 Rostock, Germany

**Keywords:** Shoulder, SLAP lesions, MEDIC, MRI, Arthroscopy

## Abstract

**Background:**

Superior labral anterior to posterior (SLAP) lesions remain a clinical and diagnostic challenge in routine (non-arthrographic) MR examinations of the shoulder. This study prospectively evaluated the ability of 3D-Multi-Echo-Data-Image-Combination (MEDIC) compared to that of routine high resolution 2D-proton-density weighted fat-saturated (PD fs) sequence using 3 T-MRI to detect SLAP lesions using arthroscopy as gold standard.

**Methods:**

Seventeen consecutive patients (mean age, 51.6 ± 14.8 years, 11 males) with shoulder pain underwent 3 T MRI including 3D-MEDIC and 2D-PD fs followed by arthroscopy. The presence or absence of SLAP lesions was evaluated using both sequences by two independent raters with 4 and 14 years of experience in musculoskeletal MRI, respectively. During arthroscopy, SLAP lesions were classified according to Snyder’s criteria by two certified orthopedic shoulder surgeons. Sensitivity, specificity, positive predictive value (PPV) and negative predictive value (NPV) of 3D-MEDIC and 2D-PD fs for detection of SLAP lesions were calculated with reference to arthroscopy as a gold standard. Interreader agreement and sequence correlation were analyzed using Cohen’s kappa coefficient.

Figure 1 demonstrates the excellent visibility of a proven SLAP lesion using the 3D-MEDIC and Fig. 2 demonstrates a false-positive case.

**Results:**

Arthroscopy revealed SLAP lesions in 11/17 patients. Using 3D-MEDIC, SLAP lesions were diagnosed in 14/17 patients by reader 1 and in 13/17 patients by reader 2. Using 2D-PD fs, SLAP lesions were diagnosed in 11/17 patients by reader 1 and 12/17 patients for reader 2. Sensitivity, specificity, PPV, and NPV of 3D-MEDIC were 100.0, 50.0, 78.6, and 100.0% for reader 1; and 100.0, 66.7, 84.6, and 100% for reader 2, respectively. Sensitivity, specificity, PPV, and NPV of 2D-PD fs were 90.9, 83.3, 90.9, and 83.3% for reader 1 and 100.0, 83.3, 91.7, and 100.0% for reader 2. The combination of 2D-PD fs and 3D-MEDIC increased specificity from 50.0 to 83.3% for reader 1 and from 66.7 to 100.0% for reader 2. Interreader agreement was almost perfect with a Cohen’s kappa of 0.82 for 3D-MEDIC and 0.87 for PD fs.

**Conclusions:**

With its high sensitivity and NPV, 3D-MEDIC is a valuable tool for the evaluation of SLAP lesions. As the combination with routine 2D-PD fs further increases specificity, we recommend incorporation of 3D-MEDIC as an additional sequence in conventional shoulder protocols in patients with non-specific shoulder pain.

## Background

Superior labrum anterior to posterior (SLAP) lesions are lesions of the attachment of the long biceps tendon and the adjacent anterosuperior to posterosuperior labral attachment to the glenoid bone, collectively forming the SLAP complex [1, 2]. The overall incidence of SLAP lesions ranges from 6 to 12% in the overall population, and reaches 28–32% in certain risk groups such as overhead athletes or military personnel [3–5]. If untreated or undiagnosed, SLAP lesions may contribute substantially to patient morbidity through prolonged shoulder pain, shoulder instability, loss of strength, and restricted shoulder movements [6]. Thus, timely diagnosis of SLAP lesions is crucial. Both the clinical and radiological diagnosis of SLAP lesions remain challenging due to non-specific clinical presentation, small size, complexity of the SLAP complex, and various normal variants of the anterosuperior and superior labrum [7]. Gold standard for the evaluation of SLAP lesions is arthroscopy, but imaging is routinely performed prior to this invasive diagnostic and therapeutic measure. Although direct MRI arthrography (MRA) is currently regarded as the gold standard of imaging for the evaluation of SLAP lesions, because its reported sensitivity and specificity is generally higher than those of conventional (without performing an arthrogram) MRI [8]. In a general radiology setting conventional MRI protocols prevail in the vast majority of shoulder MRI examinations [9] despite the fact that MRI sensitivities for detecting SLAP lesions may be as low as 19% in non-fellowship trained radiologists and 46% in fellowship trained radiologists when performing conventional 2D shoulder MRI [10].

In addition, a recent survey among the members of the European Society of Skeletal Radiology (ESSR) revealed that MRA examinations account for only 5% of all musculoskeletal MRI examinations [9]. Thus, adjustments to conventional non-MRA imaging protocols that increase diagnostic performance in detecting SLAP lesions are desirable.

The use of 3D sequences instead of or in addition to 2D sequences at the shoulder has theoretical advantages including greater resolution, thinner slices, and the ability to reformat imaging stacks in any desired orientation potentially leading to higher conspicuity of small lesions. Nevertheless, equivocal results have been reported [8, 11–14]. Moreover, different contrasts (gradient echo [GRE] compared to turbo-spin-echo based sequences) may enhance lesion detection [7].

The three-dimensional Multi Echo Data Image Combination Sequence (3D-MEDIC) is a gradient echo sequence specifically designed for musculoskeletal imaging purposes, but this sequence has not been extensively assessed. 3D-MEDIC may be useful in the diagnosis of fibrocartilaginous and ligamentous pathologies due to its high intrinsic signal-to-noise ratio (SNR) and the high resolution of a three-dimensional data stack [13, 15]. At 1.5 T, 2D-MEDIC in combination with another 3D GRE sequence has been proposed for the evaluation of fibroligamentous structures at the shoulder [7].

The diagnostic performance of 3D-MEDIC in the detection of SLAP lesions has yet to be systematically evaluated, and a comparison to arthroscopy as a gold standard is lacking. Therefore, the purpose of this study was to prospectively determine the diagnostic performance of 3D-MEDIC compared to standard 2D-PD fs in the evaluation of SLAP lesions, with reference to arthroscopy.

## Methods

### Patients

The study was approved by the institutional review board of the University of Heidelberg (S-081/2010) and performed according to the Declaration of Helsinki in its present form. Informed consent was obtained from all patients after the purpose of this study and the examination had been explained. Adult patients with chronic non-specific shoulder pain were referred to the shoulder section of the orthopedic department of the University Hospital with an indication for shoulder arthroscopy. These patients were consecutively referred to our department for presurgical MRI assessment. We excluded patients with acute traumatic injuries, known or suspected malignant diseases, patients with radiographically diagnosed osteoarthritis as well as patients that had underwent shoulder surgery before. None of the patients was sent to us with a known or distinct suspicion of a SLAP lesion. Moreover, all patients with contraindications against MRI (e.g. cardiac pacemakers) were not considered. Following the application of the in- and exclusion criteria a total of 19 patients were enrolled in this study. Two patients that underwent arthroscopy did not receive the MR study protocol and only received routine 2D MR imaging; these patients were therefore excluded from the further analysis. The final study group included 17 patients (11 males, 6 females, mean age 51.6 years ±14.77, range 22–67 years).

### MRI protocol

All images were obtained using a 70 cm open-bore 3 T whole-body MR scanner (MAGNETOM Verio, Siemens Healthineers, Erlangen, Germany) with an 18-channel total imaging matrix (TIM [102 × 18] configuration) and a 4-channel transmit-receive flex coil (Siemens Healthineers). Patients were positioned supine, head first, with external rotation of the ipsilateral arm and the flex coil in the isocenter of the magnet. All patients underwent a routine MRI shoulder protocol including high-resolution 2D-PD fs sequences. The protocol was extended by an additional 3D-MEDIC sequence. Table 1 presents technical details of the study sequences. Only the axial and coronal planes of the 2D-PD fs as well as the axial and coronal reconstructions (with 1.0 × 1.0 × 1.0 mm voxel size) of the 3D-MEDIC were used for further analysis, while all other acquired sequences and planes were neither considered nor evaluated. Radiographers were advised to stabilize the shoulder in order to prevent movement artefacts.

**Table 1:** MRI parameters of the study sequences

	3D-MEDIC	Ax 2D-PD fs	Cor 2D-PD fs
TR (ms)	41	3300	3400
TE (ms)	22	47	47
Flip angle (degree)	12	120	120
Matrix	288 × 320	346 × 384	346 × 384
Voxel size (mm)	0.6 × 0.6 × 1.0	0.6 × 0.5 × 3.0	0.6 × 0.5 × 3.0
Multiplanar reconstruction	axial, coronal, sagittal (1.0 × 1.0 × 1.0 mm)		
Field of view (mm)	180 × 200	200 × 200	200 × 200
Slice thickness (mm)	1.0	3	3
Spacing (mm)	–	3.3	3.3
Bandwidth (Hz/Pixel)	136	200	200
Echo train length	1	8	8
iPAT	GRAPPA	–	–
PAT factor	2	–	–
Acquisition time	05:17	03:32	03:38

*PD* Proton density, *fs* fat saturated, *3D-MEDIC* three dimensional Multi Echo Data Image Combination, *TSE* Turbo spin echo, *TE* Echo time, *TR* Repetition Time, *ms* milliseconds, *mm* millimeter, *Hz* Hertz, *iPAT* integrated Parallel Acquisition Techniques, *GRAPPA* Generalized Autocalibrating Partially Parallel Acquisition

**Fig. 1:**
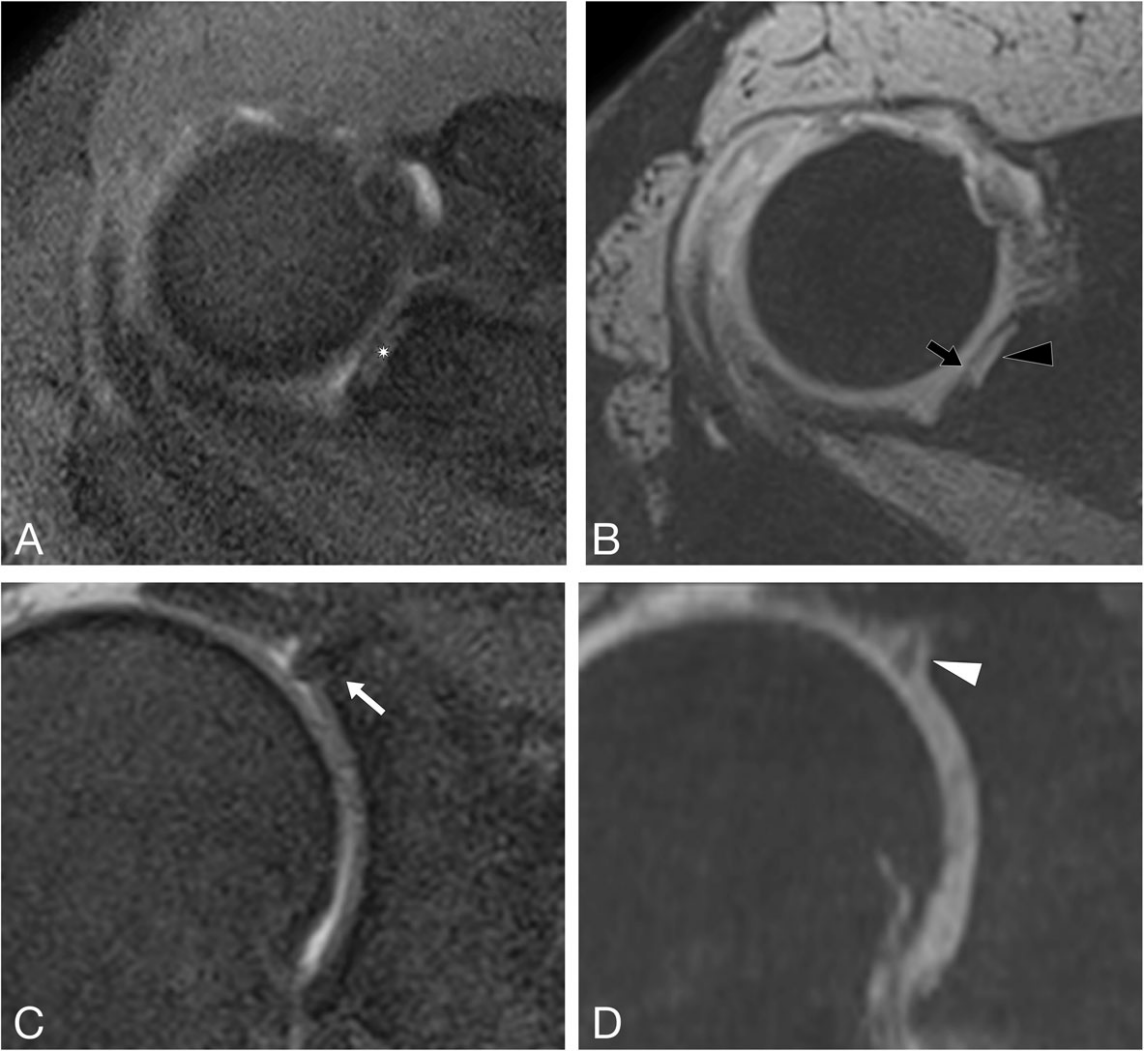
Increased visibility of SLAP lesions using 3D-MEDIC compared 2D-PD fs. Axial (**a**) and coronal (**c**) 2D-PD fs images, and axial (**b**) and coronal (**d**) reconstructions of the 3D-MEDIC of a 57-year-old male patient with an arthroscopically proven SLAP lesion. The SLAP lesion is better depicted in axial and coronal 3D-MEDIC as a high intensity line with excellent contrast to the labrum and glenoid (black arrow and white arrowhead) and a small labral discontinuity (black arrow). Consequently, both readers diagnosed the SLAP lesion using 3D-MEDIC. The signal in the axial 2D-PD fs is less pronounced (white asterisk), and only a very thin and hardly detectable hyperintense line is present in the coronal 2D-PD fs (white arrow). The less experienced Rater 1 did not diagnose a tear, while Rater 2 correctly diagnosed the SLAP lesion.

### Image analysis

All acquired images were reviewed by two independent readers with 4 (F.W.) and 14 (C.R.) years of professional experience in musculoskeletal imaging using a picture archiving and communication system (Centricity PACS, Version 4.0, GE Healthcare IT Solutions, Barrington, IL) and FDA cleared diagnostic monitors (EIZO RadiForce RX211, Eizo Corporation, Hakusan, Japan). Both 3D-MEDIC and 2D-PD fs images were evaluated separately and binarily for the presence or absence of SLAP lesions according to diagnostic criteria published in the literature (Table 2) [1]. Both radiologists were in control over slice selection, magnification, and level of windowing. They were blinded to the results of the arthroscopy procedure and the clinical data of the patients. The ambient light was kept at a minimum during the reading session. To reduce learning bias, both readers were blinded to patients’ names. Sets of 2D-PD fs sequences and 3D-MEDIC multiplanar reconstructions (MPR) were presented separately in random order. Following the completion of image evaluation and statistical analysis, discrepant false positive and false negative cases were thoroughly reevaluated in consensus to reveal possible sources of error.

**Table 2:** MR imaging criteria of SLAP lesions

Diagnostic Criteria for SLAP Lesions	
Laterally curved, linear signal in the labrum on coronal images	
Multiple or branching lines of high signal intensity in the superior labrum on coronal images	
Paralabral cyst formation extending from the superior labrum	
Full-thickness detachment with irregularly marginated high signal intensity	
Wide separation (> 2 mm) between labrum and glenoid on coronal images

**Fig. 2:**
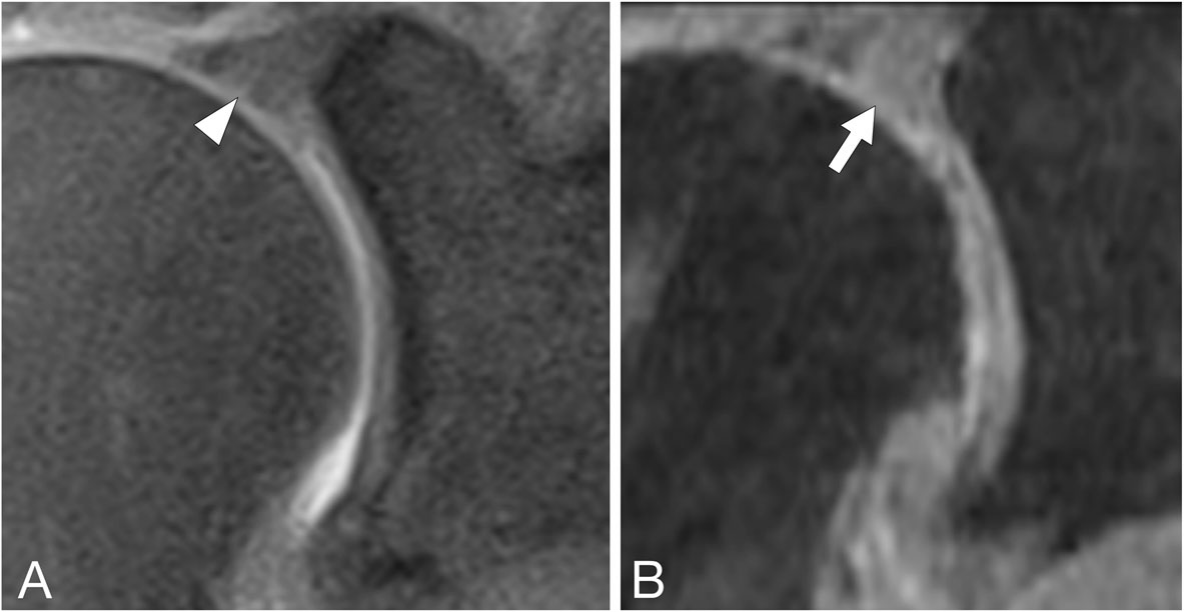
3D-MEDIC false positive case. Coronal 2D-PD fs (**a**) and coronal reconstruction of the 3D-MEDIC (**b**) of a 52-year-old patient with no SLAP lesion at arthroscopy. An increased signal within the superior labrum is appreciated in the 2D-PD fs (arrow head in A) corresponding to mucoid degeneration without a discernable tear. Neither rater noticed a tear in that sequence. The high internal signal of the labrum is even more pronounced in the 3D-MEDIC (arrow in B) with an inhomogeneous pattern. Both raters diagnosed a SLAP lesion using the 3D-MEDIC in this case.

### Shoulder arthroscopy

Shoulder arthroscopy was performed by two experienced consultant orthopedic surgeons (F.Z. and M.M.). The mean time interval between MRI examination and shoulder arthroscopy was 3.7 days (range 0–6 days). All arthroscopies were performed with the patients placed in beach-chair position and under general intravenous anesthesia. A posterior approach was used for a diagnostic inspection of the shoulder joint. If a surgical intervention was necessary, a ventral approach was established. During surgery, the SLAP complex was evaluated according to the classification of Snyder using a standardized questionnaire [16]. For statistical analysis, SLAP lesions were categorized binarily, and SLAP 1–4 lesions were summarized as SLAP lesions being present.

### Statistical analysis

Statistical analysis was performed using the statistical package SAS for Windows Version 9.4 (SAS Institute Inc., North Carolina, United States of America) and R Version 3.5.1 (www.cran.r-project.org). Demographics were analyzed descriptively. Continuous variables (age) were summarized using mean, standard deviation, median, minimum, and maximum. Qualitative variables (gender) were analyzed by calculating frequencies and percentages. The diagnostic performance of 3D-MEDIC and 2D-PD fs for SLAP lesions was described using estimates and exact 95% confidence intervals for sensitivity, specificity, positive predictive value (PPV), and negative predictive value (NPV). The statistics were calculated separately for both raters. To determine the diagnostic performance of a combination of the 3D-MEDIC and the 2D-PD fs sequence lesions of the SLAP complex were only interpreted as SLAP lesions if diagnosed in both sequences. The results of this approach were compared to the arthroscopic findings to calculate the diagnostic values of the combination of 3D-MEDIC and 2D-PD fs. To quantify the interreader agreement of 3D-MEDIC and 2D-PD fs, Cohen’s kappa coefficient was calculated and interpreted according to the classification of Landis and Koch [17]. Kappa was interpreted as slight agreement when values were between 0 and 0.2, fair when values were greater than 0.2 to 0.4, moderate when values were greater than 0.4 to 0.6, substantial when values were greater than 0.6 to 0.8, and near perfect when values were greater than 0.8 to 1.0 [17]. Moreover, Cohen’s kappa coefficient was used to determine the agreement between the 3D-MEDIC and the 2D-PD fs for each reader. Results of the comparison between were interpreted according to the classification of Landis and Koch [17].

## Results

### Diagnosis of SLAP lesions

During shoulder arthroscopy, SLAP lesions were diagnosed in 11/17 patients (76.5%). According to Snyder’s criteria, 8 out of 11 SLAP lesions were classified as Type I SLAP lesions (72.7%), one lesion was classified as Type II SLAP lesion (9.1%), and two lesions were classified as Type III lesions (18.2%). Type IV SLAP lesions were not present in our study group.

Reader 1 diagnosed SLAP lesions in 14/17 (82.4%) patients using 3D-MEDIC. All 11 lesions identified during arthroscopy were detected, with three false positive lesions that did not correspond to a tear at arthroscopy. Using 2D-PD fs, reader 1 identified SLAP lesions in 11/17 (64.7%) patients with one false positive and one false negative case. Reader 2 identified SLAP lesions in 13/17 (76.5%) patients using 3D-MEDIC. All arthroscopy-proven lesions were detected, with two false positive cases. Using 2D-PD fs, reader 2 diagnosed SLAP lesions in 12/17 (70.6%) patients. All proven SLAP lesions were detected with one false positive case. Reader 1 diagnosed type I SLAP lesions in 11 of 14 diagnosed SLAP lesions. The remaining three SLAP lesions were categorized as type II SLAP lesions. Reader 2 diagnosed type I lesions in 10 out of 13 diagnosed SLAP lesions and three type II lesions. Neither reader 1 nor reader 2 diagnosed type 3 or 4 SLAP lesions. All false-positive cases were classified as type I SLAP lesions by both readers.

### Diagnostic performance of 3D-MEDIC and 2D-PD fs

Table 3 depicts the diagnostic performance values of 3D-MEDIC and 2D-PD fs for both, reader 1 and reader 2 with reference to shoulder arthroscopy. Using the 3D-MEDIC, sensitivity and NPV of both readers were higher compared to the respective diagnostic values of the 2D-PD fs. The specificity of 3D-MEDIC was lower compared to the specificity of the 2D-PD fs. Table 4 shows the diagnostic performance of the combination of both imaging techniques. The combination of both imaging sequences leads to an increase of specificity while maintaining a high sensitivity leading to 100% sensitivity and specificity for the experienced reader.

**Table 3:** Performance of 3D-MEDIC and 2D-PD fs with reference to shoulder arthroscopy

	Sensitivity	Specificity	PPV	NPV
3D-MEDIC – reader 1	100.0% (71.5–100.0%)	50.0% (11.8–88.2%)	78.6% (49.2–95.3%)	100.0% (29.2%-100.0)
3D-MEDIC – reader 2	100.0% (71.5–100.0%)	66.7% (22.3–95.7%)	84.6% (54.6–98.1%)	100.0% (39.8–100.0%)
2D-PD fs – reader 1	90.9% (58.7–99.8%)	83.3% (35.9–99.6%)	90.9% (58.7–99.8%)	83.3% (35.9–99.6%)
2D-PD fs – reader 2	100.0% (71.5–100.0%)	83.3% (35.9–99.6%)	91.7% (61.5–99.8%)	100.0% (47.8–100.0%)

*3D-MEDIC* three dimensional Multi Echo Data Image Combination, *PD* Proton density, *fs* fat saturated, *PPV* Positive predictive value, *NPV* Negative predictive value. 95% confidence interval is depicted in parentheses.

**Table 4:** Performance of the combination of 3D-MEDIC and 2D-PD fs with reference to shoulder arthroscopy

	Sensitivity	Specificity	PPV	NPV
3D-MEDIC and 2D-PD fs positive for SLAP lesion – reader 1	90.9% (58.7–99.8%)	83.3% (35.9–99.6%)	90.9%(58.7–99.8%)	83.3% (35.9–99.6%)
3D-MEDIC and 2D-PD fs positive for SLAP lesion– reader 2	100.0% (71.5–100.0%)	100.0% (54.1–100.0%)	100.0% (71.5–100.0%)	100.0% (54.1–100.0%)

*3D-MEDIC* three dimensional Multi Echo Data Image Combination, *PD* Proton density, *fs* fat saturated, *PPV* Positive predictive value, *NPV* Negative predictive value. 95% confidence interval is depicted in parentheses.

### Interreader agreement and sequence correlation

Interreader agreement, as measured by Cohen’s kappa coefficient, was 0.82 (95% confidence interval, 0.49 to 1.00) for the 3D-MEDIC sequence and 0.87 (95% confidence interval, 0.64 to 1.00) for the 2D-PD fs sequence, resembling near perfect agreement according to Landis and Koch [17]. Cohen’s kappa between 3D-MEDIC and 2D-PD fs was 0.56 (95% confidence interval, 0.16 to 0.97) for reader 1 and 0.55 (95% confidence interval, 0.10 to 1.00) for reader 2, which corresponded to a moderate agreement according to Landis and Koch [17].

## Discussion

The diagnosis of SLAP lesions is a clinical and radiological challenge due to the heterogenous clinical presentation of affected patients as well as the complexity and size of the SLAP complex. MRA is currently regarded as the gold standard of imaging for the detection of SLAP lesions. However, the vast majority of MRI examinations of the shoulder are performed as conventional, non-contrast enhanced MR exams [9]. Additionally, most shoulder MRI exams are conducted in outpatient facilities and interpreted by radiologists without fellowship training in musculoskeletal imaging, contributing to a lower sensitivity for the detection of SLAP lesions [10]. Therefore, improvements in conventional imaging protocols to facilitate diagnosis are warranted. In our study, we evaluated a 3D-MEDIC sequence for its ability to detect SLAP lesions of the shoulder and compared its performance to 2D-PD fs of conventional imaging protocols. The results were correlated with those of subsequent shoulder arthroscopy as a gold standard. Compared to shoulder arthroscopy, 3D-MEDIC imaging yielded an excellent sensitivity of 100% and an excellent NPV of 100% for the detection of SLAP lesions for both experienced and less experienced readers. With regards to the less experienced reader, sensitivity of 3D-MEDIC was even higher than the sensitivity of conventional 2D-PD fs. In other words, 3D-MEDIC imaging was able to detect all lesions and excluded SLAP lesions regardless of the experience of the reading radiologist. In a single case, a SLAP lesion confirmed subsequently was only detectable on 3D-MEDIC but not on 2D-PD fs sequences, contributing to the increased visibility. However, this occurred at a cost of moderate to high-moderate specificity with three false positive diagnoses for the less experienced reader and two false positive diagnoses for the experienced reader. The combination of 3D-MEDIC and 2D-PD fs sequences of the conventional protocol proved to be beneficial. Compared to the diagnostic performance of 3D-MEDIC alone, the diagnostic performance of combined 3D-MEDIC and 2D-PD fs led to an increase in specificity for both readers. Moreover, the experienced reader reached excellent performance values (sensitivity, specificity, PPV, and NPV) using the combined methods. Several possibilities may explain the excellent sensitivity of 3D-MEDIC. MEDIC is a T2* weighted gradient-echo sequence specifically designed for musculoskeletal and neuroradiological purposes and combines up to six echoes in a single image leading to a higher signal-to-noise ratio and reduced susceptibility [13, 15, 18]. Compared to other 3D sequences at the wrist, 3D-MEDIC exhibits a high contrast and signal-to-noise ratio as well as the best visibility of fibrocartilaginous and ligamentous tissue [13]. At 7 T a MEDIC sequence exhibited a high level of anatomical detail with regards to the labrum as well as good labrum/fluid contrast at the hip [19]. Furthermore, the continuous 3D slice acquisition down to 1 mm slice thickness leads to reduction of partial volume artefacts, thereby increasing the visibility of smaller lesions. Moreover, the acquisition of a 3D image stack with an isotropic voxel size of 1.0 × 1.0 × 1.0 mm allows high-resolution multiplanar reconstructions in any desired plane to further increase the conspicuity of smaller lesions [12, 20]. One fact that limits routine 3D imaging of the shoulder is the longer acquisition time compared to that of a single 2D sequence combined with lower robustness. Some 3D sequences at the shoulder have acquisition times of up to 9.5 min [12]. The 3D-MEDIC acquisition time in our study was about 5 min and included the entire shoulder joint. The short acquisition time and robustness of the sequence itself may have contributed to the fact that all examinations and reconstructions in the present study were evaluable. As 3D-MEDIC itself has not been systematically evaluated with regards to SLAP lesions, there is a paucity of literature for comparing our findings. Lee et al. paired GRE sequences, namely a 2D-MEDIC sequence and 3D dual echo steady-state (DESS), and compared both sequences against a combination of conventional T1-weighted spin echo (SE) sequences at 1.5 T with respect to glenoid labral tears including superior, anterior, and posterior labral tears [7]. With arthroscopy as a reference, the combination of both GRE sequences showed a higher sensitivity of 88% compared to that of T1 SE combination [7], in line with our findings. However, the use of a combination of two different GRE sequences, lower field strengths, different assessment, and the use of a 2D instead of a 3D-MEDIC limits the comparability of their findings to our results. Pahwa et al. used a 3D-MEDIC sequence to evaluate the fibroligamentous structures at the wrist and compared the results to those of conventional MRI and MRI arthrography. They reported a higher sensitivity of 3D-MEDIC for detecting tears of the triangulate fibrocartilage complex and ligamentous structures of the wrist compared to that of conventional 2D-PD fs sequences with open surgery or arthroscopy as reference. This is in line with our results, although the comparability is limited by differences in the field strength and joint of interest [21]. Nevertheless, the findings of Pahwa et al. and Lee et al. as well as our own findings support the assumption that 3D-MEDIC sequence has high sensitivity for detecting fibroligamentous pathologies. The diagnostic performance of conventional MRI for the detection of SLAP lesions has been evaluated in various studies and meta-analyses. In two recent meta-analyses, the pooled sensitivity for the detection of SLAP lesions was estimated to be between 63 to 76% [22, 23]. However, in several studies, the sensitivity of conventional MRI was as low as 38 to 46% [10, 22–24]. Indeed, Connolly et al. reported a sensitivity as low as 19% for the detection of SLAP lesions by radiologists without fellowship training in musculoskeletal radiology [10]. As stated above, the vast majority of shoulder examinations are performed using a conventional MRI protocol and conducted in outpatient facilities [9]. In relation to the aforementioned published sensitivities concerning SLAP diagnosis in conventional MRI, the need for improvements of conventional MRI protocols is evident. The sensitivity of 3D-MEDIC in our study was higher than the pooled sensitivity published in a meta-analysis of conventional MRI. A careful reevaluation of the false positive cases led to the assumption that severe degenerative or mucoid changes within the labrum with a non-homogenous increase in internal signals of the labrum itself may resemble tears or at least limit diagnostic certainty. In line with this assumption, Loredo et al. evaluated two gradient-echo sequences in a cadaveric study of the glenoid labrum and observed changes of the labral signal intensity which were histopathologically attributed to mucoid or eosinophilic degeneration, calcification, ossification, fibrovascular tissue, synovial tissue or a combination of these [25]. Therefore, 3D-MEDIC seems to be sensitive to changes in the fibrocartilage matrix [25, 26]. For cases with severe degenerative changes of the labrum in particular, the thorough evaluation of 2D-PD fs sequences may add diagnostic confidence. The interreader agreement measured by Cohen’s kappa for the detection of SLAP lesions was 0.82 for the 3D-MEDIC and 0.87 for the PD fs sequences, consistent with an almost perfect agreement according to Koch and Landis [17]. This indicates the reproducibility of the findings using both sequences which is crucial for reliable image interpretation.

There are several limitations to our study. First, our study sample was small, and the results regarding diagnostic performance have to be verified in further (multi-centric) studies with larger patient cohorts. Furthermore, we were unable to perform subgroup analysis according to different subtypes of SLAP lesions due to the small sample size. In addition, the patient cohort was recruited in a tertiary referral center and was therefore highly selected. Hence, the results may not be generalizable to the general population. Moreover, we only focused on the diagnosis of SLAP lesions. It has been described that SLAP lesions are common findings in asymptomatic middle-aged patients [27]. Therefore, future studies are needed to evaluate the clinical impact of an improved detection of SLAP lesions in different settings.

Further, the use of GRE sequences like 3D-MEDIC may be limited in patients with metal implants, due to the increased susceptibility for artefacts [28]. As we excluded patients who undergone shoulder surgery, our findings may only be applicable to patients without prior surgery. This is of great interest as the detection of complications as well as newly acquired shoulder pathologies in a postsurgical setting is crucial for the diagnosis and the initiation of treatment measures [29]. Therefore, further studies examining the feasibility of the 3D-MEDIC in different surgical setting with metallic and non-metallic implants are necessary. Moreover, future studies are required to validate the findings of our study in larger and less selected patient cohorts. Also, studies comparing the performance of 3D MEDIC with MR arthrograms in the same patient cohort are desirable. Future studies may also investigate whether the increase in diagnostic performance leads to a change in patient treatment or patient outcome.

## Conclusions

The 3D-MEDIC sequence exhibits excellent sensitivity and NPV for the evaluation of SLAP lesions in both experienced and less experienced readers. It is a robust sequence with a high resolution and the benefits of the 3D acquisition and may be a powerful tool to confidently detect or exclude SLAP lesions. Thus, we recommend the addition of this sequence to routine conventional shoulder protocols in patients with non-specific shoulder pain. As the combination with routine 2D-PD fs increases specificity while maintaining high sensitivity, we recommend employing both sequences to achieve the greatest diagnostic confidence.

## Data Availability

The datasets used and/or analyzed during the current study are available from the corresponding author on reasonable request.

## Abbreviations

ESSR: European Society of Musculoskeletal Radiology
GRE: Gradient echo
MEDIC: Multi-echo-data-image-combination
MPR: Multiplanar reconstructions
MRA: MRI arthrography
NPV: Negative predictive value
PD fs: Proton-density weighted fat-saturated
PPV: Positive predictive value
SLAP: Superior labral anterior to posterior
SNR: Signal-to-noise ratio
